# Effects of Various Extents of High-Frequency Hearing Loss on Speech Recognition and Gap Detection at Low Frequencies in Patients with Sensorineural Hearing Loss

**DOI:** 10.1155/2017/8941537

**Published:** 2017-12-27

**Authors:** Bei Li, Yang Guo, Guang Yang, Yanmei Feng, Shankai Yin

**Affiliations:** Department of Otolaryngology Head and Neck Surgery, Shanghai Jiao Tong University Affiliated Sixth People's Hospital, No. 600, Yishan Road, Xuhui District, Shanghai 200233, China

## Abstract

This study explored whether the time-compressed speech perception varied with the degree of hearing loss in high-frequency sensorineural hearing loss (HF SNHL) individuals. 65 HF SNHL individuals with different cutoff frequencies were recruited and further divided into mildly, moderately, and/or severely affected subgroups in terms of the averaged thresholds of all frequencies exhibiting hearing loss. Time-compressed speech recognition scores under both quiet and noisy conditions and gap detection thresholds within low frequencies that had normal thresholds were obtained from all patients and compared with data from 11 age-matched individuals with normal hearing threshold at all frequencies. Correlations of the time-compressed speech recognition scores with the extents of HF SNHL and with the 1 kHz gap detection thresholds were studied across all participants. We found that the time-compressed speech recognition scores were significantly affected by and correlated with the extents of HF SNHL. The time-compressed speech recognition scores also correlated with the 1 kHz gap detection thresholds except when the compression ratio of speech was 0.8 under quiet condition. Above all, the extents of HF SNHL were significantly correlated with the 1 kHz gap thresholds.

## 1. Introduction

In ENT clinical, patients with high-frequency sensorineural hearing loss (HF SNHL) always complain about the intelligibility of the fast speech. Sensorineural hearing loss (SNHL) is very commonly encountered in the clinic. Researches showed that compared to the normal-hearing (NH) individuals, the ability to comprehend speech in noise decreased in the SNHL individuals [1–3]. Low-intensity signals masked in speech cannot be perceived by those with SNHL, rendering poor speech recognition. Although speech contains a wide range of frequencies [4], according to Ardoint and Lorenzi [5], the most important frequency range in terms of speech perception is 1-2 kHz. Usually, the SNHL begins at high frequencies and slowly spreads to lower frequencies. Once SNHL extends into the low-frequency region (1-2 kHz), the speech recognition ability of SNHL individuals becomes even worse.

Of all those with SNHL, even individuals with only HF SNHL usually complain about the intelligibility of fast speech, especially in noise. Accumulated evidence shows that the speech recognition scores of HF SNHL patients with normal low-frequency hearing are poorer than those of NH individuals, even when speech stimuli are limited to low frequencies [6–10]. Age also played an important role in speech perception [11]. Leigh-Paffenroth and Elangovan [12] found significant poorer temporal processing in the low-frequency regions (with normal thresholds) in middle-aged individuals even without HF SNHL, compared to the younger individuals. Fullgrabe et al. [13] found declines in speech perception in older persons compared to the youth persons, even the audiometric sensitivities of both were within normal ranges. It is necessary to exclude the influence of age and hearing differences in low-frequency region to study the impact of HF SNHL in speech perception in low frequencies. After auditory sensitivity and age were controlled, research suggested that suprathreshold temporal processing deficits did exist [6, 14]. Others showed that noise-induced HF SNHL affected low-frequency temporal resolution in guinea pigs, even though the thresholds in the low-frequency region were within normal ranges [15, 16]. In this point of view, the speech perception difficulties that many SNHL individuals experienced probably consist of not only SNHL of the high-frequency region but also the temporal processing disability in the low-frequency region.

Previous studies found that speech recognition ability varied among individuals with different extents of SNHL [12, 17–20]. Andrade et al. [18] reported that the speech recognition thresholds correlated with the extents of SNHL in individuals with nonflat audiograms. Also, self-assessed scores of hearing disability were associated with the pure-tone thresholds [17, 19, 20]. Notably, Dobie [19] explored the relationships between pure-tone averages (at 0.5, 1, 2, and 3 kHz) and self-assessed hearing disability scores of 1001 patients and found no correlation between self-assessed scores and pure-tone averages in patients whose pure-tone averages were below 25 dB HL. However, a linear correlation was evident between the self-assessed hearing disability scores and pure-tone averages in patients whose pure-tone averages were above 25 dB HL [19].

However, whether and how HF SNHL affects low-frequency speech perception and temporal resolution remains largely unknown. In the present work, we grouped patients by cutoff frequency (1, 2, and 4 kHz) of HF SNHL. Thus, the thresholds at and below each cutoff frequency were within normal ranges, and the thresholds beyond the cutoff frequencies were higher than 25 dB HL. And gap detection tasks were used to evaluate the temporal resolution of low-frequency region. Speech recognition scores upon delivery of time-compressed sentences under both quiet and noisy conditions and gap detection thresholds were measured and compared between HF SNHL groups with the same cutoff frequency but various degrees of HF SNHL and age-matched NH group.

## 2. Materials and Methods

### 2.1. Participants

A total of 76 individuals were recruited, including 65 HF SNHL patients and 11 NH individuals. All HF SNHL participants were recruited from the Department of Otolaryngology Head and Neck Surgery at Shanghai Jiao Tong University Affiliated Sixth People's Hospital, and NH individuals from the staff of the same hospital. No neurological, psychiatric, or other disorders that would undermine speech recognition ability were identified in all participants including the HF SNHL participants. The program was approved by the Ethics Committee of Shanghai Jiao Tong University Affiliated Sixth People's Hospital. All participants gave written informed consent prior to study commencement.

All participants were native Mandarin-speaking Chinese. All NH individuals had pure-tone thresholds 25 dB HL or less at all octave frequencies between 250 and 8000 Hz, in both ears. HF SNHL patients were rigorously selected according to the following criteria: (1) symmetrical SNHL, with threshold differences of 15 dB or less (at all frequencies) between both ears for more than 6 months; (2) pure-tone thresholds of 25 dB HL or less, both at and below the cutoff frequencies; (3) pure-tone thresholds > 25 dB HL above the cutoff frequencies; and (4) type A or Ad type tympanograms.

**Figure 1:**
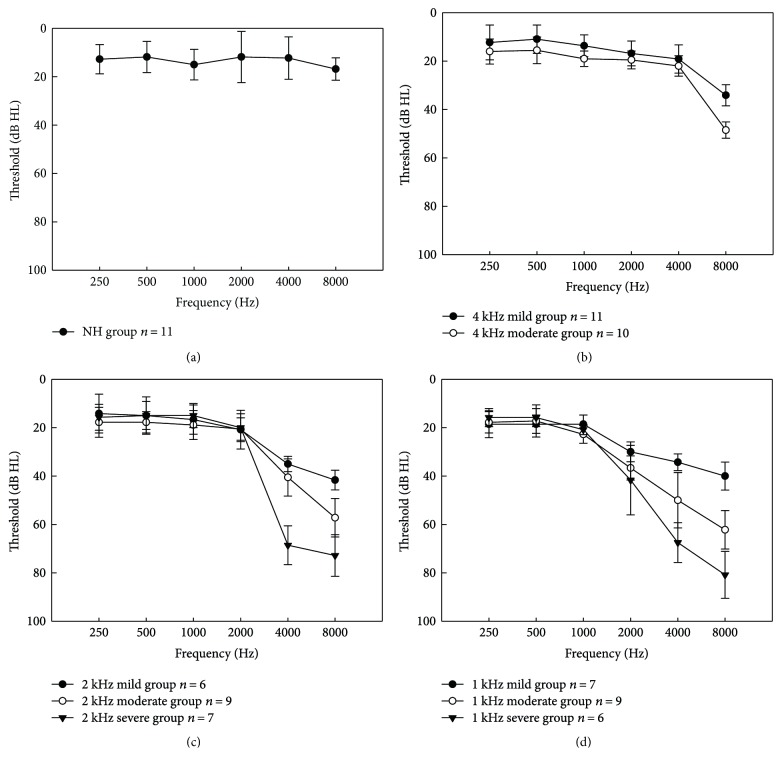
Mean audiometric thresholds (dB HL), with standard deviations, for each group. The audiometric thresholds of the tested ears for the normal hearing (NH) and 4, 2, and 1 kHz HF SNHL groups are shown in (a), (b), (c) and (d), respectively.

HF SNHL patients were grouped by the HF SNHL cutoff frequencies evident on audiograms (e.g., 1, 2, and 4 kHz). In each of these three groups, patients were further subdivided into those with mild (25–40 dB HL), moderate (41–60 dB HL), and severe (>60 dB HL) HF SNHL subgroups, defined by the means of averaged pure-tone threshold across frequencies higher than the cutoff frequency. Thus, finally, we formed eight HF SNHL groups, including mild, moderate, and severe groups with cutoff frequencies at 1 and 2 kHz and mild and moderate groups with cutoff frequency at 4 kHz, and one NH group. The means and standard deviations of the auditory thresholds of the tested ears for all groups are shown in Figure 1. Demographic data of all groups are shown in Table 1.

### 2.2. Stimuli and Procedure

The gap detection task was measured in a three-interval forced-choice procedure. For the gap marker, white noise was low-pass filtered at cutoff frequencies of 1, 2, and 4 kHz, respectively, via 3000th-order finite impulse response filter with an approximately −116 dB/octave filter slope.

In brief, a three-interval forced-choice program had been run on MATLAB software (version 7.0). Three buttons were presented on a monitor to the participant who was asked to indicate which one of the three stimuli was different (i.e., which of the three stimuli was inserted with a gap). As each of the three stimuli playing, the corresponding button was highlighted in red (from left to right). Participant was instructed to click one of the three buttons with the mouse as a response after each presentation of three signals. The next trial was initiated after an answer was given. All subjects were trained to be familiar with the procedure before formal test. The training would last until their performances reached platforms, respectively. No feedback was given to the subject throughout the test. The gap varying in size from 20 to 1 ms was embedded in the middle of one of the three noise bursts (total duration: 1000 ms for each). The gap was shaped using a 1 ms, raised cosine envelope. Each test, commenced with a gap of 20 ms, was followed by a down sequence (in 2 ms steps) until the first erroneous answer was recorded. The two-down, one-up procedure was then adopted (with a gap step size of 1 ms) until the appointed reversals were reached. In gap detection tests, the frequency spectra of the gap markers tested in the HF SNHL groups differed. For example, 4 kHz group members were tested separately with 1, 2, and 4 kHz gap markers. Those of the 2 kHz groups were tested using 1 and 2 kHz gap markers. For those of the 1 kHz groups, only the 1 kHz gap marker test was tested.

Speech perception was assessed using the Mandarin version of the Hearing in Noise Test (MHINT) of the House Ear Institute [21], representing a daily and communicative style of speech, which could be easily understood by native Mandarin-speaking listeners with various degrees of education. Speech was time-compressed using Praat software (version 5.3), without any significant change in the power spectrum [22]. We used three compression ratios: 0.6, 0.8, and 1.0 that of the normal speech rate (the compression ratio of 1.0, namely, was normal speech rate). Speech recognition tests were run under both quiet and noisy [signal-to-noise ratio (SNR): −5 dB] conditions.

All test signals were presented at 75 dB SPL under both quiet and noisy conditions and were delivered monaurally through Sennheiser HD580 headphones. Only right ears were tested, and a 40 dB SPL speech-shaped noise was conducted to the left ears as masker all along the tests. To create noisy conditions, a speech-shaped noise of the same spectrum as that of the MHINT sentence was presented with SNR at −5 dB. The noise began 500 ms before the sentence and continued for 500 ms after the sentence had concluded. A complete set of tests required approximately 30 min. Practice was conducted before each test, and feedback was provided. After practice, each participant achieved stable recognition scores. During a speech recognition test, each sentence was played only once, and no feedback was given. The same methods were also applied by Feng et al. [14].

## 3. Results

### 3.1. Age Matching and Pure-Tone Thresholds of the NH and HF SNHL Groups

One-way analysis of variance (ANOVA) showed that the mean ages of all nine groups did not differ significantly (*F*_(8,75)_ = 1.097, *p* = 0.376).

Comparisons of the averaged thresholds across the frequencies with normal thresholds in all groups showed that the thresholds of frequencies exhibiting normal hearing did not differ significantly among the groups (*F*_(8,75)_ = 1.899, *p* = 0.075).

### 3.2. Gap Detection Task

**Figure 2:**
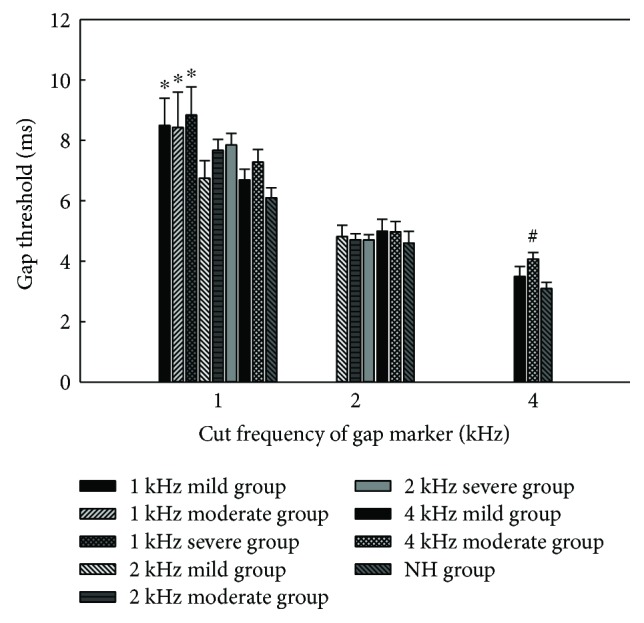
The mean gap thresholds in the high-frequency sensorineural hearing loss (HF SNHL) groups and normal-hearing (NH) group. The error bars indicate standard errors. ∗ indicates gap thresholds of the 1 kHz mild, moderate, and severe HF SNHL groups with 1 kHz gap marker which were significantly higher than those of the NH group and the 4 kHz mild HF SNHL group; # indicates gap thresholds of the 4 kHz moderate HF SNHL group which were significantly higher than those of the NH group for 4 kHz gap marker.

The gap thresholds of groups varying in terms of gap marker cutoff frequency are shown in Figure 2. The gap thresholds of the gap markers with different cutoff frequencies for the same listener group were compared firstly. Paired *t*-tests showed that the gap thresholds of 1 kHz gap marker were significantly higher than those of 2 kHz gap marker for 2 kHz mild HF SNHL group (*t* = 5.349, *p* = 0.003), 2 kHz moderate HF SNHL group (*t* = 10.639, *p* < 0.001), and 2 kHz severe HF SNHL group (*t* = 7.22, *p* < 0.001). One-way repeated ANOVA showed significant main effects of cutoff frequencies of gap marker on gap thresholds of 4 kHz mild HF SNHL group (*F*_(2,20)_ = 19.334, *p* < 0.001), 4 kHz moderate HF SNHL group (*F*_(2,18)_ = 21.063, *p* < 0.001), and NH group (*F*_(2,20)_ = 57.133, *p* < 0.001); the post hoc analyses (LSD tests) revealed that gap thresholds of 1 kHz gap marker, 2 kHz gap marker, and 4 kHz gap marker differed from each other significantly for the three groups, respectively. Generally, the gap thresholds of all groups gradually decrease as cutoff frequencies of the gap marker increase gradually.

Then, data derived from different groups with the same gap marker frequency were analyzed by one-way ANOVA. There was a significant difference when the cutoff frequency of gap marker is 1 kHz (*F*_(8,75)_ = 2.189, *p* = 0.039); the post hoc analysis (LSD test) revealed that the gap thresholds of the NH group and 4 kHz mild HF SNHL group were significantly lower than those of the 1 kHz mild HF SNHL group, 1 kHz moderate HF SNHL group, and 1 kHz severe HF SNHL group. And there was also a significant difference when the cutoff frequency of gap marker is 4 kHz (*F*_(2,31)_ = 3.515, *p* = 0.043); the post hoc analysis (LSD test) revealed that the gap thresholds of the NH group were significantly lower than those of the 4 kHz moderate HF SNHL group with 4 kHz gap marker. However, no difference was evident with 2 kHz gap marker (*F*_(5,53)_ = 0.231, *p* = 0.947). When presented with gap marker of the same cutoff frequency, in general, the gap thresholds of various groups tended to be higher if the range of HF SNHL was wider or the degrees of hearing impairment were higher.

### 3.3. Time-Compressed Speech Recognition

**Figure 3:**
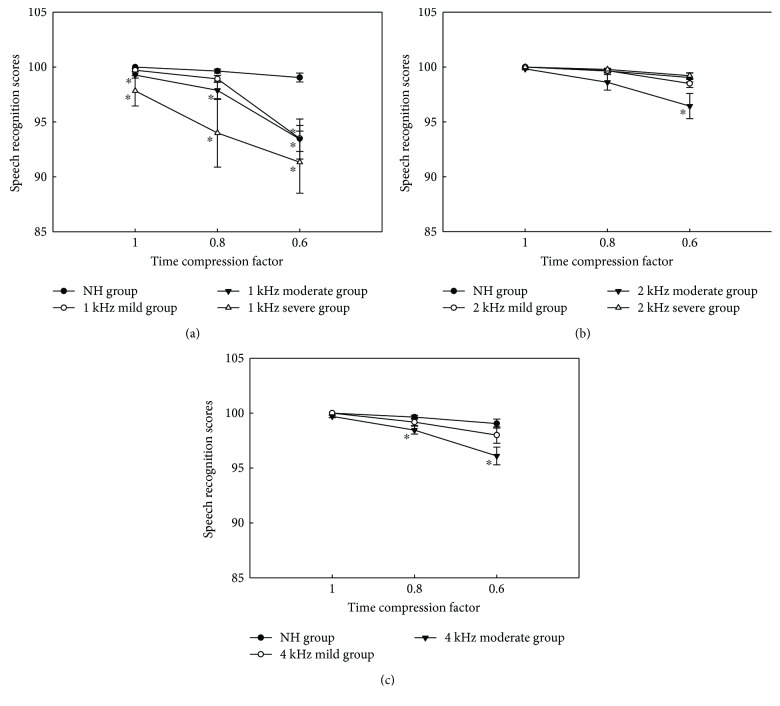
Speech recognition scores under quiet conditions for the normal-hearing (NH) and high-frequency sensorineural hearing loss (HF SNHL) groups, as a function of the time compression ratio. The scores for the 1, 2, and 4 kHz HF SNHL groups are shown in (a), (b), and (c), respectively. The error bars indicate standard errors. ∗ indicates significant difference of speech recognition scores when compared with that of the NH group at the same time compression factor.

**Figure 4:**
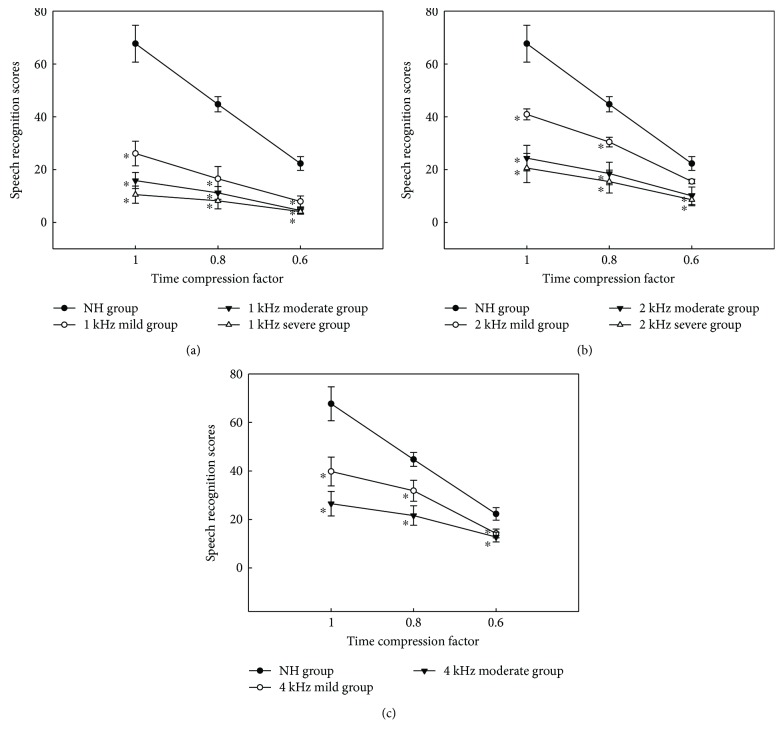
Speech recognition scores under noisy conditions (SNR = −5 dB) for the normal-hearing (NH) and high-frequency sensorineural hearing loss (HF SNHL) groups, as a function of the time compression ratio. The scores for the 1, 2, and 4 kHz HF SNHL groups are shown in (a), (b), and (c), respectively. The error bars indicate standard errors. ∗ indicates significant difference of speech recognition scores when compared with that of the NH group at the same time compression factor.

The original scores under quiet and noisy conditions are shown in Figures 3 and 4, respectively. Overall, the speech recognition scores of all groups decreased as the time compression ratio fell from 1.0 (normal speech rate) to 0.6 and the scores were lower under noisy conditions than those under quiet conditions at the same time compression ratio.

Before analysis, all speech recognition scores were arcsine-transformed to avoid ceiling or floor effects. Data from the NH and eight HF SNHL groups in quiet were subjected to two-way repeated-measures ANOVA to test the effects of group and time compression ratio on speech recognition. The effects of group and compression ratio were both significant: *F*_group(8,67)_ = 5.368, *p* < 0.001 and *F*_compression(2,134)_ = 114.028, *p* < 0.001. There was a statistically significant two-way interaction between group and time compression ratio (*F*_(16,134)_ = 2.130, *p* = 0.010). The LSD method was applied in post hoc comparisons, to explore the effect of the extent of HF SNHL on speech recognition scores in quiet. When speech compression ratio was 0.6, all HF SNHL groups scored significantly lower than the NH group except the 2 kHz mild and severe HF SNHL groups and 4 kHz mild HF SNHL group; when speech compression ratio was 0.8, the NH group scored significantly higher than the 1 kHz moderate and severe HF SNHL groups and 4 kHz moderate HF SNHL group, while the NH group scored significantly higher than the 1 kHz moderate and severe HF SNHL groups when speech compression ratio was 1.0. The differences among the 1 kHz mild, moderate, and severe HF SNHL groups were not statistically significant when speech compression ratio was 0.6 or 1.0, but the scores of the 1 kHz mild HF SNHL groups were significantly higher than the 1 kHz severe HF SNHL groups when speech compression ratio was 0.8; the differences among the 2 kHz mild, moderate, and severe HF SNHL groups and the differences between the 4 kHz mild and moderate HF SNHL groups were not statistically significant for all three speech compression ratios.

A two-way repeated-measures ANOVA was used to evaluate the effects of group and time compression ratio on speech recognition in noise. The effects of group and compression ratio were both significant: *F*_group(8,67)_ = 11.541, *p* < 0.001 and *F*_compression(2,134)_ = 144.785, *p* < 0.001. There was a statistically significant two-way interaction between group and time compression ratio (*F*_(16,134)_ = 4.434, *p* ≤ 0.001). The LSD method was used in post hoc comparisons to explore the effect of the extent of HF SNHL on speech recognition scores in noise. When speech compression ratio was 0.8 and 1.0, the differences between scores of the NH group and all HF SNHL groups were significant; when speech compression ratio was 0.6, the NH group scored significantly higher than all HF SNHL groups except the 2 kHz mild HF SNHL group. The differences among the 1 kHz mild, moderate, and severe HF SNHL groups were not statistically significant when speech compression ratio was 0.6 or 0.8, but the scores of the 1 kHz mild HF SNHL groups were significantly higher than those of 1 kHz severe HF SNHL groups when speech compression ratio was 1.0; the 2 kHz mild HF SNHL group scored significantly higher than the 2 kHz moderate and severe HF SNHL groups when speech compression ratio was 0.6 and 0.8, and the 2 kHz mild HF SNHL group scored significantly higher than the 2 kHz severe HF SNHL group when speech compression ratio was 1.0. The differences between the 4 kHz mild and moderate HF SNHL groups were not statistically significant for all three speech compression ratios.

As a whole, at the same time compression ratio, the scores of the NH group were better than those of any HF SNHL group, and the scores of those with HF SNHL decreased as the degree of HF SNHL increased, which was more obvious under noisy conditions.

### 3.4. Correlation Analysis

We explored relationships between time-compressed speech recognition scores, pure-tone HF averages, and gap detection thresholds, by calculating *Pearson* correlations as follows: (1) between pure-tone HF averages (for NH groups, the average was calculated across 2, 4, and 8 kHz; for HF SNHL groups, the average was calculated across frequencies where exhibited hearing loss) and time-compressed speech recognition scores; (2) between pure-tone HF averages and the 1 kHz gap detection thresholds; and, (3) between the time-compressed speech recognition scores and the 1 kHz gap detection thresholds (all participants underwent 1 kHz gap detection testing). As shown in Table 2, the pure-tone HF averages were significantly correlated with time-compressed speech recognition scores at compression ratios of both 0.6 and 0.8, and normal speech recognition scores (all *p* values < 0.05), under both quiet and noisy conditions. What is noteworthy is that pure-tone HF averages were significantly correlated with the 1 kHz gap detection thresholds (*Pearson* correlation = 0.367, *p* = 0.001). In Table 3, the results showed that 1 kHz gap thresholds were significantly correlated with the speech recognition scores at all compression ratios under noisy conditions (all *p* values ≤ 0.001). This was also true under quiet conditions for both normal speech and that at a 0.6 compression ratio (*p*_0.6compression_ ≤ 0.001, *p*_normal speed_ = 0.045).

## 4. Discussion

Our primary purpose in the present study was to explore whether and how HF SNHL affected time-compressed speech perception and gap detection in low-frequency region with normal auditory threshold. We found that the time-compressed speech recognition scores of the HF SNHL group were poorer than those of the NH group and decreased as the extent of HF SNHL increased in patients with the same cutoff frequency. Generally, the recognition scores of patients with severe HF SNHL were poorer than the scores of those with moderate HF SNHL, which in turn were poorer than those of patients with mild HF SNHL at the same cutoff frequencies, under both quiet and noisy conditions (Figures 3 and 4).

As shown in Table 2, pure-tone averages of HF SNHL were significantly correlated with the time-compressed speech recognition scores. These results suggested that the ability to recognize time-compressed speech was affected by HF SNHL and correlated with the extent of HF SNHL. These results are similar to those of our previous study and indeed extend our earlier work [14]. We previously showed that the time-compressed speech recognition scores of the HF SNHL subjects were poorer than those of NH individuals [14]. However, the effect of the extent of HF SNHL on speech recognition was not explored in detail. Therefore, in the present study, we focused on the effect of varying levels of HF SNHL on time-compressed speech recognition abilities.

Indeed, the extent of HF SNHL affects the ability of speech recognition. Moore [23] found that, in individuals with cochlear hearing loss of up to approximately 45 dB, a change in audibility was the single most important contributor to speech perception problems. However, when the extent of hearing loss was greater, poor discrimination of suprathreshold stimuli also became of major importance. Nimitbunnasarn et al. [24] examined tonal identification in Thai speakers with normal hearing and different extents of SNHL. Identification ability was affected by SNHL per se and the extent thereof [24]. Jerger et al. [25] explored correlations among speech recognition performance, the pure-tone hearing level, and the age of individuals with SNHL. The relationship between the extent of hearing loss and speech recognition score was strongest in older individuals with SNHL. Other studies sought correlations between self-assessed hearing inventories and individual pure-tone thresholds [12, 17, 19] or between speech reception and pure-tone thresholds [20]. All data suggested that the extents of SNHL correlated with the speech recognition scores. Our results are consistent with such findings and suggest that the extents of HF SNHL are also correlated with the time-compressed speech recognition scores. Similar hearing configurations that vary in the extent of hearing loss may impact speech recognition differently, especially when speech is fast. More severe speech disruption is evident as the severity of hearing loss rises.

It is worth noting that the pure-tone averages of HF SNHL correlated with the 1 kHz gap thresholds significantly, which indicates that the HF SNHL could impair the temporal resolution of the low-frequency region. This is in line with our previous research results, which suggests that HF SNHL exerted an off-channel effect on temporal processing ability in the low-frequency region of the auditory system, whether in guinea pigs or humans [14, 15]. This off-channel effect may contribute to the difficulty, which is experienced by patients with normal hearing in low frequencies but suffered from HF SNHL, of perceiving the time-compressed speech [14] and temporal fine structure speech [6].

The relationship between speech recognition and temporal resolution, another focus of the present study, remains unclear. Previous studies suggested that temporal resolution played an important role in speech recognition [26–29]. However, no influence of any temporal processing ability, such as gap detection, on speech perception, has been conclusively shown [11].

To determine whether the gap detection threshold correlated with time-compressed speech recognition ability, we carefully controlled for age and the extent of HF SNHL. As shown in Table 3, the gap thresholds with 1 kHz-low-pass-filtered noise were negatively correlated with the time-compressed speech recognition scores, suggesting that speech recognition ability was also affected by temporal resolution of the low-frequency region, even whose auditory sensitivity was normal.

## 5. Conclusions

Even when age was controlled, the extent of HF SNHL impacted the ability to recognize compressed speech. The greater the extent of HF SNHL was, the poorer the speech recognition ability was. The significant correlation between the extents of HF SNHL and gap detection thresholds implied that there was probable off-channel mechanism underlying. The decrease of time-compressed speech recognition ability may be partly attributable to the increased thresholds in gap detection task, which signified the deterioration of suprathresholdly temporal resolution.

## Figures and Tables

**Table 1:** Demographic data for the NH group and HF SNHL subgroups.

Group	Male	Female	Age mean ± SD (yrs)
NH	2	9	45.6 ± 13.5
4 kHz mild	5	6	51.2 ± 8.5
4 kHz moderate	5	5	48.3 ± 5.3
2 kHz mild	3	3	52.0 ± 5.4
2 kHz moderate	3	6	46.4 ± 7.9
2 kHz severe	3	4	52.9 ± 11.3
1 kHz mild	3	4	42.6 ± 8.2
1 kHz moderate	4	5	52.7 ± 13.4
1 kHz severe	3	3	51.0 ± 8.6

NH: normal hearing; SD: standard deviation.

**Table 2:** Correlation analysis between pure-tone averages of high-frequency hearing loss and speech recognition scores with different compression and different test backgrounds.

	Compression ratio of speech in quiet			Compression ratio of speech in noise		
	1	0.8	0.6	1	0.8	0.6
*Pearson* correlation	−0.279	−0.269	−0.329	−0.694	−0.627	−0.536
*p* value	0.015^∗^	0.019^∗^	0.004^∗^	<0.001^∗^	<0.001^∗^	<0.001^∗^

For NH groups, the average was calculated across 2, 4, and 8 kHz; for HF SNHL groups, the average was calculated across frequencies where exhibited hearing loss. ∗ indicates *p* values smaller than 0.05.

**Table 3:** Correlation analysis between 1 kHz gap thresholds and speech recognition scores with different compression ratios under different test backgrounds.

	Compression ratio of speech in quiet			Compression ratio of speech in noise		
	1	0.8	0.6	1	0.8	0.6
*Pearson* correlation	−0.231	−0.185	−0.427	−0.388	−0.394	−0.381
*p* value	0.045^∗^	0.109	<0.001^∗^	0.001^∗^	<0.001^∗^	0.001^∗^

∗ indicates *p* values smaller than 0.05.
